# A Novel Microspheres Formulation of Puerarin: Pharmacokinetics Study and In Vivo Pharmacodynamics Evaluations

**DOI:** 10.1155/2016/4016963

**Published:** 2016-12-29

**Authors:** Xiao Song, Xihui Bai, Shiyu Liu, Linjuan Dong, Hui Deng, Changli Wang

**Affiliations:** College of Pharmacy, Shaanxi University of Chinese Medicine, Xi'an, Shaanxi 712046, China

## Abstract

The aim of this study was to investigate the pharmacokinetics and pharmacodynamics of puerarin loaded carboxymethyl chitosan microspheres (Pue-CCMs). The differences in pharmacokinetics parameters of rats after intragastric administration of Pue-CCMs and puerarin were investigated using HPLC. To assess the protective effect of Pue-CCMs on myocardial injury in rats, serum levels of creatine kinase (CK), lactate dehydrogenase (LDH), total superoxide dismutase (T-SOD), and malondialdehyde (MDA) were measured, in addition to pathological examinations and immunohistochemical staining. Our present study has shown that the AUC_0–*t*_, *C*_max_, *T*_max_, MRT_0–*t*_ of Pue-CCMs, and puerarin were 20.176 mg·h/L, 3.778 *μ*g/mL, 1 h, 4.634 h and 9.474 mg·h/L, 2.618 *μ*g/mL, 0.542 h, and 3.241 h, respectively. Pue-CCMs alleviated myocardial ischemic injury. Pretreatment with Pue-CCMs could significantly decrease CK, LDH, and MDA levels and increase T-SOD level in the serum. Pue-CCMs downregulated expression of the Bcl-2 associated X protein (Bax) and upregulated B-cell lymphoma-2 (Bcl-2) expression. Compared with puerarin group, the Pue-CCMs group could improve the oral bioavailability of puerarin. The protective effect of Pue-CCMs against myocardial injury was significantly greater than puerarin at the same dose. In summary, Pue-CCMs should be a qualified and promising candidate as a new oral preparation of puerarin.

## 1. Introduction

Ischemic heart disease (IHD) is caused by the imbalance between coronary flow and myocardial demand due to coronary circulation and represents a significant cause of morbidity and mortality in the world [1]. Therefore, the need for a drug effective in the treatment of ischemic heart disease is apparent.

**Figure 1:**
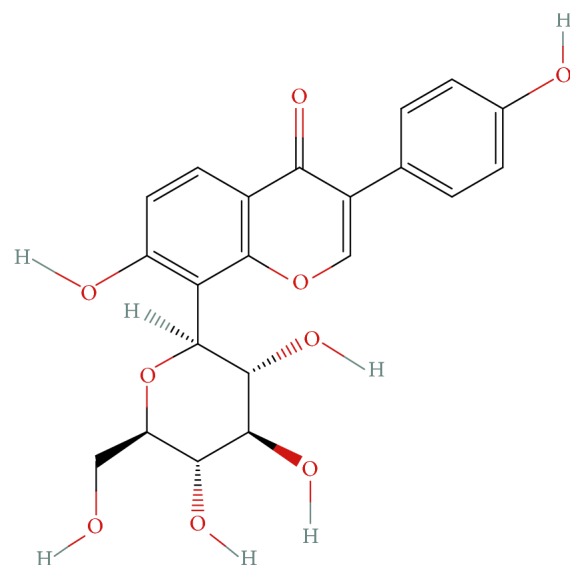
Structural formulas of puerarin.

Radix Puerariae is the root of* Pueraria lobata* (Willd.) Ohwi. Puerarin (7,4′-dihydroxyisoflavone-8*β*-glucopyranoside, Figure 1) is a major active ingredient of Radix Puerariae [2, 3]. A large number of pharmacological studies have indicated that puerarin has a protective effect upon the cardiovascular system. Therefore, it is commonly used in the treatment of coronary heart disease, high blood pressure, and related diseases [4–6].

Due to poor water solubility and low oral bioavailability, intravenous injection remains the main drug delivery method [7–9]. However, due to the short elimination half-life in human beings, it is necessary to administer frequent or high doses, which leads to severe side effects and restricts its clinical application [10, 11]. Thus, oral formulation with improved absorption of puerarin has attracted widespread attention.

Chitosan is a promising candidate of drug delivery systems because of its nontoxicity, high biocompatibility, biological adhesion, and biodegradability [12]. However, chitosan only can be dissolved in acidic conditions, which limits its application [13]. Carboxymethyl chitosan, a water soluble derivative of chitosan, can drastically increase the solubility of chitosan at neutral pH values without affecting its characteristic properties [14]. For these advantages, carboxymethyl chitosan has received much attention for its potential applications to drug delivery systems.

In the early study, the preparation of glutaraldehyde cross-linked, Pue-CCMs was optimized by central composite design-response surface methodology [15]. In this study, we evaluated the pharmacokinetic parameters of puerarin in normal rats after intragastric administration of a single dose of Pue-CCMs in comparison to the performance of puerarin under the same conditions. Secondly, we investigated the protective effect of Pue-CCMs and puerarin against myocardial injury under the same dose and conducted preliminary study on the mechanisms through which Pue-CCMs protect against myocardial injury.

## 2. Materials and Methods

### 2.1. Chemicals and Reagents

The puerarin standard (purity ≥ 98%, Batch no. 110752-201514) was purchased from the National Institutes for Food and Drug Control (Beijing, China). Pue-CCMs were obtained through self-made preparation in the laboratory. Puerarin (purity of 98%) was purchased from Shaanxi Xu Ang Biological Technology Co. Ltd. (Shaanxi, China). Chromatographic-grade methanol was obtained from Thermo Fisher Scientific (USA). All other chemicals and reagents were of analytical grade, and the water was deionized and double-distilled.

ISO was purchased from Nanjing Senbeijia Biological Technology Co. Ltd. (Nanjing, China). Ethylurethanm was purchased from Shanghai Shan Pu Chemical Co. Ltd. (Shanghai, China). Propranolol was purchased from Shaanxi Yongshou Pharmaceutical Co. Ltd. (Shaanxi, China). Creatine kinase (CK), lactate dehydrogenase (LDH), total superoxide dismutase (T-SOD), and the malondialdehyde (MDA) test kit were all purchased from Nanjing Jiancheng Biological Engineering research institute (Nanjing, China). B-cell lymphoma-2 (Bcl-2) antibodies and Bcl-2 associated X protein (Bax) antibodies were both purchased from Wuhan Boshide Biological Technology Company (Wuhan, China).

### 2.2. Experimental Animals

Healthy male Sprague-Dawley (SD) rats (weight: 250 ± 20 g) were obtained from the Animal Experiment Center of Xi'an Jiao Tong University (Xi'an, Shaanxi, China). The animals were maintained under standard laboratory conditions (temperature 25 ± 2°C, humidity 60 ± 5%, 12/12 h light/dark cycle) for one week prior to experiments. The rats fasted overnight but were supplied with water ad libitum prior to the experiments. This study was approved by the Animal Ethical Committee of Shaanxi University of Chinese Medicine.

### 2.3. Preparation Method and the Characteristics of the Pue-CCMs

Pue-CCMs were prepared by emulsion cross-linking method. The 0.5 g carboxymethyl chitosan was dissolved in 20 mL distilled water, and 0.5 g puerarin was added to the above solution. The polymer solution containing puerarin was added drop wise into 120 mL liquid paraffin containing 6% span-80 resulting in formation of W/O emulsion. The 1 mL glutaraldehyde was added drop wise into the W/O emulsion and continues to mix to form microspheres. Finally, the Pue-CCMs were gained by centrifugation at 2700 ×g for 5 min and washed several times with petroleum and dried for 12 h in a vacuum oven at 60°C. The drug loading, encapsulation efficiency, and particle size of Pue-CCMs were 25.73%, 51.47% and 78.8 *μ*m, respectively.

### 2.4. Pharmacokinetics and Bioavailability Studies

#### 2.4.1. Experimental Conditions

The analysis was performed using the HPLC system (Thermo Fisher Scientific, USA). Analytes were separated on a Thermo Hypersil GOLD C_18_ column (4.6 mm × 250 mm, 5 *μ*m). The mobile phase system consisted of methanol (A) and 1% acetic acid water (B) with the following program (v/v): 21% A at 0–30 min. The wavelength of the UV detector was set at 250 nm for puerarin with a flow rate of 1.0 mL/min and a column temperature of 30°C.

#### 2.4.2. Sample Preparation

In this study, the protein precipitation method was used to extract puerarin. The process is as follows: an aliquot of 100 *μ*L thawed plasma sample was transferred into an EP tube, 400 *μ*L of a mixture of methanol and acetonitrile (90 : 10, V/V) was added to precipitate protein, and samples were vortex for 3 min (Vortex-genie2, Gene Company Limited, USA) and centrifuged at 9700 ×g for 5 min. The supernatant was transferred into another EP tube and evaporated to dryness under N_2_ (Nitrogen Evaporators MG-2200, Tokyo Rikakikai Co. Ltd., Japan). After 100 *μ*L of methanol was added to redissolve, the sample was vortex for 5 min and centrifuged at 9700 ×g for 5 min. The supernatant was injected into the HPLC system for analysis.

#### 2.4.3. Method Validation

Specificity was assessed by analyzing blank plasma, blank plasma samples spiked with puerarin standard at the LLOQ level, and rat plasma samples after administration of Pue-CCMs, puerarin, and puerarin injection.

Linearity was tested at seven plasma concentration levels in the range of 0.53–53 *μ*g/mL (0.53, 1.06, 2.65, 5.3, 10.6, 26.5, and 53 *μ*g/mL). The calibration curve was obtained by plotting the peak area of puerarin versus the puerarin concentration. The lower limit of quantification (LLOQ) was calculated based on a signal-to-noise ratio of 10 : 1.

Precision and accuracy were determined by analyzing in five replicates of 1.06, 5.3, and 26.5 *μ*g/mL on the same day and on five consecutive days. Accuracy was expressed as the relative error (RE%) and precision was assessed by calculating the relative standard deviation (RSD%).

The extraction recovery was determined by comparing the peak area obtained from the extracted spiked sample with that of the postextracted spiked sample at the corresponding concentration.

The short- and long-term stabilities were evaluated by analyzing plasma samples of puerarin kept at room temperature for 24 h and in the freezer (−20°C) for one month. The freeze-thaw stability was carried out by detecting samples undergoing three freeze (−20°C) - thaw (room temperature) cycles.

#### 2.4.4. Drug Administration

Eighteen rats were randomly divided into the following 3 groups: the Pue-CCMs group, the puerarin group, and the puerarin injection group. Animals in the Pue-CCMs group were administered 2 g/kg of Pue-CCMs (i.g., equivalent to 500 mg/kg of puerarin). Animals in the puerarin group were administered 500 mg/kg of puerarin (i.g.). Those in the puerarin injection group were administered 20 mg/kg of puerarin (i.v.). After dosing, the rats were anesthetized with small amounts of ether. Approximately 0.5 mL of blood was collected from the canthus vein plexus of rats at predetermined time points (0, 0.167, 0.333, 0.5, 0.75, 1, 1.5, 2, 4, 6, 8, 10, and 12 h for Pue-CCMs group; 0, 0.167, 0.333, 0.5, 0.75, 1, 1.5, 2, 4, 6, 8, and 10 h for puerarin group; and 0.08, 0.17, 0.33, 0.5, 0.75, 1, 1.5, 2, and 4 h for puerarin injection group) and put into clean heparinized EP tubes. Blood samples were immediately centrifuged at 9700 ×g for 10 min (Centrifuge-X1, Gene Company Limited, USA), and plasma samples were stored at −20°C until analysis.

#### 2.4.5. Statistical Analysis

The pharmacokinetic parameters were calculated through noncompartmental analysis based on the DAS3.2.8 pharmacokinetic program (invented by the Clinical Trial Center of Shanghai University of Traditional Chinese Medicine, Shanghai, China), including the area under the concentration-time curve from time zero to the last sampling time point (AUC_0–*t*_), area under the concentration-time curve from time zero to infinity (AUC_0–*∞*_), maximum plasma concentration of puerarin (*C*_max_), time to maximum plasma concentration of puerarin (*T*_max_), elimination half-life (*T*_1/2_), mean residence time (MRT), total clearance (Cl), and the volume of distribution at terminal state (Vz).

The calculation formula of the bioavailability of puerarin is as follows:

Absolute bioavailability:
  (1) $$\begin{array}{c} F\left(\;\%\;\right)=\left[\;\frac{{AUC}_{0\text{–}t,i.g.}\times {Dose}_{i.v.}}{{AUC}_{0\text{–}t,i.v.}\times {Dose}_{i.g.}}\;\right]\times 100\%. \end{array}$$
Relative bioavailability:
  (2) $$\begin{array}{c} F\left(\;\%\;\right)=\frac{{AUC}_{0\text{–}t,Pue-CCMs}}{{AUC}_{0\text{–}t,puerarin}}\times 100\%. \end{array}$$

Data are presented as the mean ± SD. The differences were analyzed by* t*-test using IBM SPSS Statistics 19.0 program. *P* < 0.05 was considered statistically significant.

### 2.5. Pharmacodynamics Studies

#### 2.5.1. Experiment Protocol

Thirty rats were randomly allocated into five groups as follows: control group, model group, positive control group, Pue-CCMs group, and puerarin group. The Pue-CCMs groups was given microspheres (2 g/kg/d, equivalent to 500 mg/kg/d of puerarin), the puerarin group was given puerarin suspension (500 mg/kg/d), and the positive control group was administered propranolol (20 mg/kg/d). The control group and model group were both given physiological saline. All treatments involved intragastric administration. Rats were pretreated for 7 days and make model with ISO (80 mg/kg, except for the control group) via subcutaneous injection on 5th, 6th, and 7th days. After 30 min following the last dose of ISO administration, rats were weighed and anesthetized with an intraperitoneal injection of 20% ethylurethanm solution (1.0 g/kg). A midline incision was made in the abdomen. Blood samples were collected into polypropylene tubes from the abdominal aortas of the rats. After blood collection, rat hearts were excised, rinsed in ice-cold isotonic saline, removed, and fixed in 10% formalin solution. Blood samples were immediately centrifuged at 2700 ×g for 10 min, and serum samples were transferred into EP tubes and stored at −20°C until analysis.

#### 2.5.2. Histopathological Examinations

The heart tissue was processed for sectioning and staining by standard histological methods. Sections from the left ventricle were stained with hematoxylin-eosin (HE). A light microscope (DP-73, OLYMPUS, Japan) at 400x magnification was used to examine each slide.

#### 2.5.3. Determination of CK, LDH, T-SOD, and MDA in the Serum

T-SOD, CK, and LDH levels were measured using commercial enzyme-linked immunosorbent assay. MDA levels were measured using spectrophotometrical diagnostic kits. All measurements were performed according to the kit manufacturers' instructions.

#### 2.5.4. Immunohistochemical

Protein expression levels of Bcl-2 and Bax were examined using the immunohistochemical method. A light microscope at 400x magnification was used to examine each slide.

#### 2.5.5. Statistical Analysis

Statistical differences were analyzed by* t*-test using IBM SPSS Statistics 19.0 program. Results were expressed as the mean ± SD and differences were considered statistically significant at a value of *P* < 0.05.

## 3. Results

### 3.1. Pharmacokinetics

#### 3.1.1. Method Validation

**Figure 2:**
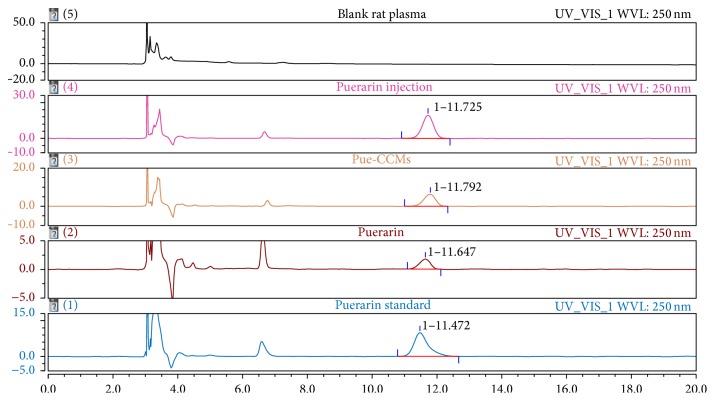
HPLC chromatograms of specificity investigation.

Typical chromatograms are shown in Figure 2. As is shown in the figure, the endogenous impurity does not interfere with the determination of puerarin, which demonstrates that the method is specific.

The regression equation of the calibration curve for puerarin is *Y* = 0.4603*X* + 0.0136, *R*^2^ = 0.9997. The LLOQ for puerarin is 0.53 *μ*g/mL.

The results of precision and accuracy quantification are shown in Table 1. The results indicate that the method was precise and accurate for the determination of puerarin in rat plasma.

The recoveries of high, medium, and low concentrations of puerarin were 85.03%, 84.64% and 86.65%, respectively. The results show that the method used for sample preparation was satisfactory.

The stability results for puerarin in rat plasma are summarized in Table 2. Puerarin was stable in rat plasma when stored at room temperature for 24 h and in the freezer (−20°C) for one month and stable after three freeze-thaw cycles in rat plasma.

#### 3.1.2. Pharmacokinetic Data Analysis

**Figure 3:**
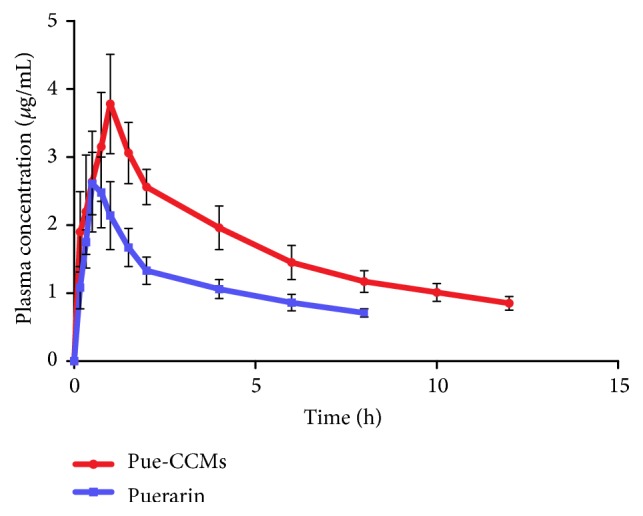
Mean plasma concentration-time curves of puerarin after single dose intragastric administration of microspheres and puerarin suspension in rats (*n* = 6).

**Figure 4:**
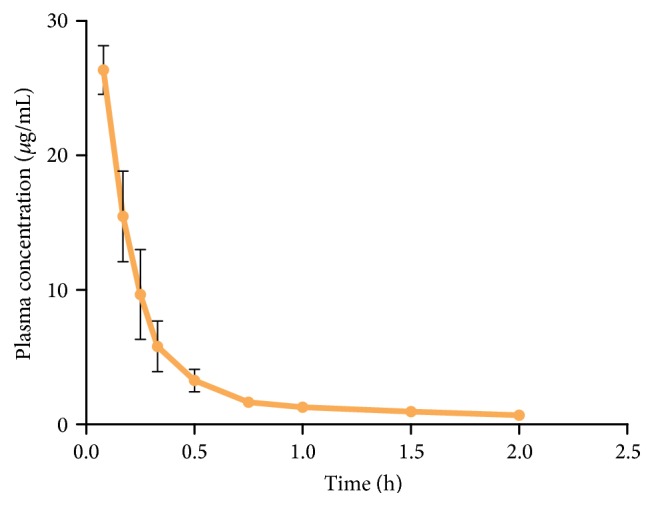
Mean plasma concentration-time curves of puerarin after intravenous injection in rats (*n* = 6).

The mean plasma concentration-time profiles are presented in Figures 3 and 4. Pharmacokinetic parameters of puerarin were determined using noncompartmental analysis and summarized in Table 3. The results show that the AUC_0–*t*_ and *C*_max_ values of Pue-CCMs group were significantly higher than puerarin group (20.176 ± 3.23 versus 9.474 ± 1.27 mg·h/L; 3.778 ± 0.73 *μ*g/mL versus 2.618 ± 0.48 *μ*g/mL, *P* < 0.01). The *T*_max_ and MRT_0–*t*_ values of Pue-CCMs group were significantly longer than puerarin group (1 ± 0 h versus 0.542 ± 0.09 h; 4.634 ± 0.14 h versus 3.241 ± 0.12 h, *P* < 0.01). Compared with the puerarin group, the Cl and Vz values were significantly lower in the Pue-CCMs group (*P* < 0.01). According to the formula, the absolute bioavailability of Pue-CCMs was 8.96%, and the relative bioavailability was 212.96%.

### 3.2. Pharmacodynamics

#### 3.2.1. Myocardial Tissue Pathology

**Figure 5:**
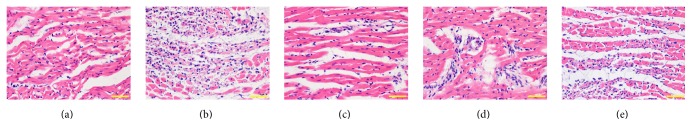
Effects of Pue-CCMs on histopathological changes in rat hearts stained with H&E. Representative sections (magnification: 40×) are from the hearts of control (a), model (b), propranolol (c), Pue-CCMs (d), and puerarin (e).

HE-stained images showed that cardiomyocytes in the control group (Figure 5(a)) were arranged in an orderly manner, whereas the model group (Figure 5(b)) revealed marked infiltration of inflammatory cells and loss of striations with nuclear changes. The positive control group (Figure 5(c)) showed clear striations and a small amount of inflammatory cell infiltration. Compared with model group, Pue-CCMs group displayed a significant decrease in the area of infiltrating inflammatory cells (Figure 5(d)), while the puerarin group did not display a significant decrease (Figure 5(e)).

#### 3.2.2. Effects of Pue-CCMs on CK, LDH, T-SOD, and MDA Serum Levels

**Figure 6:**
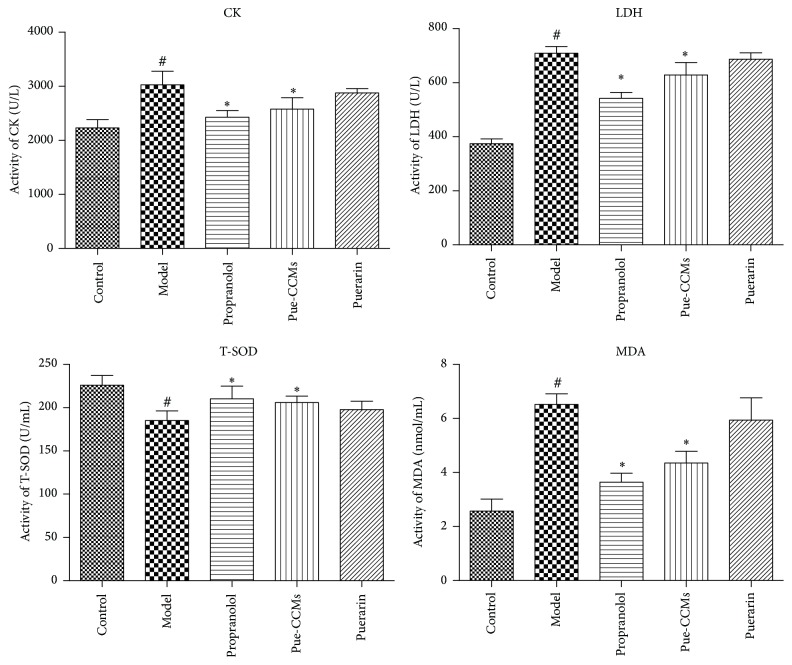
Effect of Pue-CCMs on creatine kinase (CK), lactate dehydrogenase (LDH), total superoxide dismutase (T-SOD), and malondialdehyde (MDA) levels in serum. Values are expressed as mean ± SD (*n* = 6). ^#^*P* < 0.01 versus control group; ^*∗*^*P* < 0.01 versus model group.

Compared with the control group, CK, LDH, and MDA levels increased and SOD level decreased significantly in the model group (^#^*P* < 0.01). Pretreatment with Pue-CCMs significantly increased SOD level and decreased CK, LDH, and MDA levels in comparison to the model group (^*∗*^*P* < 0.01, Figure 6).

#### 3.2.3. Effects of Pue-CCMs on Bcl-2 and Bax Expressions in Myocardial Tissue of Rats with Myocardial Injury

**Figure 7:**
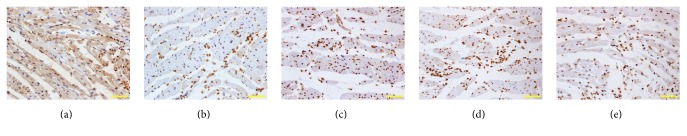
Effects of Pue-CCMs on Bcl-2 expression in rat hearts stained immunohistochemically. Representative sections (magnification: 40×) are from the hearts of control (a), model (b), propranolol (c), Pue-CCMs (d), and puerarin (e).

**Figure 8:**
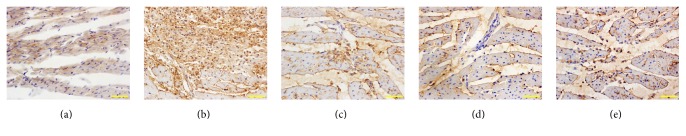
Effects of Pue-CCMs on Bax expression in rat hearts stained immunohistochemically. Representative sections (magnification: 40×) are from the hearts of control (a), model (b), propranolol (c), Pue-CCMs (d), and puerarin (e).

From Figures 7 and 8 and Table 4, the expression of Bcl-2 and Bax in each group can be observed. Compared with control group, the model group displayed significantly decreased levels of Bcl-2 and increased levels of Bax. The Pue-CCMs group displayed significantly increased levels of Bcl-2 and decreased levels of Bax in comparison to the model group.

## 4. Discussion

In recent years, the puerarin oral preparation has received the attention of researchers. The researchers used different methods to prepare puerarin oral preparation, for example, puerarin microemulsion and nanoparticles [5, 16]. At present, most of the researchers have studied the pharmacokinetics of puerarin oral preparation; however, there are few studies on pharmacodynamics of puerarin oral preparation. Therefore, this study not only studies the pharmacokinetics of Pue-CCMs but also makes a preliminary evaluation on the pharmacodynamics of Pue-CCMs.

Related studies have shown that CMCS displays characteristics of biological adhesion and penetration. As a drug carrier, it can prolong the drug retention time in the body and improve the bioavailability of drugs. The main mechanism is the following two aspects: first, the amino group and carboxyl group in the CMCS molecule can react with glycoprotein in mucus to form hydrogen bonds, which produce its adhesive characteristics. Secondly, carboxymethyl chitosan enhances the permeability of intestinal epithelia by opening the tight junctions between cells, thereby favoring paracellular drug transport [17]. In this study, we compared the pharmacokinetic parameters of puerarin in normal rats after single dose intragastric administration of Pue-CCMs and puerarin. Compared with the puerarin group, the AUC_0–*t*_, *C*_max_, *T*_max_, and MRT_0–*t*_ of the Pue-CCMs group were increased significantly, and the Cl and Vz were significantly decreased. Through the calculation, we found that the absolute bioavailability of Pue-CCMs was 8.96%, and the relative bioavailability was 212.96%. The results of pharmacokinetics study show that Pue-CCMs may delay the release of puerarin and increase the oral bioavailability of puerarin.

Animal models of simulating human ischemic heart disease have made considerable contributions in the treatment and prevention of ischemic heart disease. Isoproterenol (ISO) is a synthetic catecholamine and a *β*-adrenergic receptor agonist, which leads to biochemical and structural changes that cause myocardial injury. ISO-induced myocardial injury is considered as one of the most widely used experimental model. However, some studies indicated that estrogen can protect the heart from ischemic injury; this means that estrogen would have affected the experimental results if we use the female rats [18]. So we used the male rats to build the acute myocardial ischemia induced by ISO in this study.

Growing evidence indicates that reactive oxygen species (ROS) play an important role in cardiovascular diseases and that the accumulation of ROS in mitochondria can lead to apoptotic cell death [19]. The Bcl-2 family is a major regulator of mitochondrial permeability, including proapoptotic proteins and antiapoptotic proteins. The proapoptotic protein Bax triggers the release of proapoptotic factors into the cytoplasm, thereby inducing cell apoptosis; on the other hand, Bcl-2 forms heterodimers with proapoptotic proteins and inhibits cell apoptosis [20].

Puerarin has already been proven to exhibit potent free-radical scavenging activity due to its antioxidant properties [21]. In this study, Pue-CCMs were demonstrated to aid in the alleviation of myocardial ischemic injury. Pretreatment with Pue-CCMs decreased CK, LDH, and MDA levels and increased T-SOD level in the serum. Pue-CCMs downregulated expression of the proapoptotic protein Bax and upregulated Bcl-2 expression. The results indicate that Pue-CCMs produced an effect superior to that of puerarin suspension at the same dose, and protective effect of Pue-CCMs may be related to its antioxidant effects.

However, there are still some deficiencies in this study. Future investigations are necessary to determine the pharmacokinetics of microspheres in the pathological model of rats and the antioxidant molecular mechanism of Pue-CCMs in vitro.

## 5. Conclusions 

In summary, the Pue-CCMs should be considered a qualified and promising candidate in a new oral preparation of puerarin.

## Figures and Tables

**Table 1:** Precision and accuracy of the method for the determination of puerarin in rat plasma (*n* = 5).

Concentration (*μ*g/mL)	Intraday		Interday	
	Precision RSD%	Accuracy RE%	Precision RSD%	Accuracy RE%
1.06	3.60	−3.54	5.05	−4.47
5.3	5.79	−5.45	4.36	−4.38
26.5	1.15	2.72	1.32	2.78

**Table 2:** Stability of puerarin in rat plasma under different conditions (*n* = 5).

Concentrations (*μ*g/mL)	Room temperature RSD%	Freeze-thaw cycles RSD%	Long-term stability RSD%
1.06	4.49	6.30	8.48
5.3	3.41	3.29	7.90
26.5	0.86	1.44	3.21

**Table 3:** Pharmacokinetic parameters of different groups (mean ± SD, *n* = 6).

Parameters	Unit	Microsphere	Puerarin	Puerarin injection
AUC_0−*t*_	mg·h/L	20.176 ± 3.23^*∗*^	9.474 ± 1.27	9.01 ± 0.79
AUC_0−*∞*_	Mg·h/L	30.423 ± 3.98^*∗*^	16.428 ± 2.38	9.928 ± 0.88
*C*_max_	*μ*g/mL	3.778 ± 0.73^*∗*^	2.618 ± 0.48	26.348 ± 1.81
*T*_max_	h	1 ± 0^*∗*^	0.542 ± 0.09	0.08 ± 0
*T*_1/2_	h	8.356 ± 1.84	6.84 ± 1.3	0.951 ± 0.12
MRT_0−*t*_	h	4.634 ± 0.14^*∗*^	3.241 ± 0.12	0.336 ± 0.02
Cl	L/h	16.659 ± 2.06^*∗*^	30.991 ± 4.63	2.027 ± 0.16
Vz	L	199.879 ± 44.65^*∗*^	302.386 ± 51.58	2.772 ± 0.33

Comparison between the microspheres and puerarin group; ^*∗*^*P* < 0.01; ^#^*P* < 0.05.

**Table 4:** The IOD values of Bcl-2 and Bax (mean ± SD, *n* = 6).

group	Bcl-2	Bax
Control	0.0262 ± 0.0015	0.0199 ± 0.0025
Model	0.0151 ± 0.0015^#^	0.0434 ± 0.0022^#^
Positive control	0.0236 ± 0.0010^*∗*^	0.0232 ± 0.0018^*∗*^
Pue-CCMs	0.0206 ± 0.0014^*∗*^	0.0361 ± 0.0021^*∗*^
Puerarin	0.0170 ± 0.0094	0.0431 ± 0.0012

^#^ *P* < 0.01 versus control group; ^*∗*^*P* < 0.01 versus model group.
